# *In Silico* Pharmacokinetic Profile and Antimalarial Efficacy of 3‐Chloro‐4‐(4‐Chlorophenoxy)Aniline

**DOI:** 10.1155/bri/6509347

**Published:** 2026-06-02

**Authors:** Milka W. Waithera, Shadrack K. Kimani, Stephen Njoroge, Thomas Nyahanga, Johnson K. Kinyua

**Affiliations:** ^1^ Department of Biochemistry, School of Biomedical Sciences, Jomo Kenyatta University of Agriculture and Technology, Nairobi, Kenya, jkuat.ac.ke; ^2^ Centre for Research in Infectious Diseases, Mount Kenya University, Thika, Kenya, mku.ac.ke; ^3^ Department of Biological and Physical Sciences, School of Pure and Applied Sciences, Karatina University, Karatina, Kenya, karu.ac.ke

**Keywords:** ADMETox prediction, drug resistance, malaria, new antimalarial compounds, *Plasmodium falciparum*

## Abstract

Despite the urgent need for new therapies to treat multidrug‐resistant virulent *Plasmodium falciparum*, many promising antimalarial candidates fail to progress to clinical use because their pharmacokinetic (PK) profile is poorly characterized. This study predicted the PK profile of a new compound, 3‐chloro‐4‐(4‐chlorophenoxy)aniline (ANI), in silico using the pkCSM online platform. In vitro cytotoxicity in Vero E6 cells and antiplasmodial activity against chloroquine‐sensitive (*P. falciparum* 3D7) and chloroquine‐resistant (*P. falciparum* W2) strains were screened using the MTT colorimetric and the [^3^H]‐hypoxanthine incorporation assays, respectively. In vivo antimalarial efficacy was evaluated in *Plasmodium berghei* ANKA and piperaquine‐resistant (PQ^R^) *P. berghei* parasites in mouse models employing early and established infection tests. *In silico* predictions show that ANI is a substrate for CYP 3A4 and CYP 2D6 and is unlikely to inhibit the hERG potassium ion channel. ANI exhibited low cytotoxicity, with a CC_50_ value of 7.90 × 10^2^ ± 86.40 μM, and potent antiplasmodial activity, with IC_50_ values of 1.72 ± 0.09 μM and 1.84 ± 0.21 μM against the 3D7 and W2 strains, respectively. The selectivity index of greater than 400 indicates that ANI has a broad safety margin. In vivo, ANI demonstrated antimalarial efficacy with ED_50_ values of 3.07 mg/kg/day against *P. berghei* ANKA and 2.81 mg/kg/day against PQ^R^ *P. berghei* parasites. Notably, mice treated with ANI in the established infection model survived for up to 30 days without observable adverse effects. These findings highlight ANI as a potential antimalarial compound and support further drug evaluation and development.

## 1. Introduction

Malaria is still a significant impediment to the advancement of public health in most malaria‐endemic countries, particularly in remote settings experiencing limited access to healthcare facilities. In 2024, there were an estimated 282 million malaria cases and 610,000 deaths globally, representing an increase in both morbidity and mortality compared to the previous year [1]. The WHO African Region bore a disproportionate malaria burden, accounting for 95% of global cases (265 million) and deaths (579,000), with children under five years comprising about 75% of these deaths [1]. The human malaria parasites have consistently developed resistance to almost all available antimalarial drugs [2]. Recent studies confirm increasing global threats of *Plasmodium falciparum* resistance to the artemisinin‐based combination therapies (ACTs), demonstrated by a delay in parasite clearance after the standard 3‐day treatment course, particularly in malaria‐endemic regions of Southeast Asia and Sub‐Saharan Africa [3]. The present situation has placed a huge demand for the search of alternative novel malaria drugs. Major efforts have been directed toward the discovery of alternative compounds such as pure derivatives of natural products, for instance, alstonine and himbeline [4]; synthetic antibacterial derivatives, for instance, those comprising tetracyclines such as eravacycline [5]; and the synthesis of hybrid antimalarial drugs such as artesunate‐3‐chloro‐4‐(4‐chlorophenoxy)aniline [2].

*De novo* drug discovery and development are multistage, extremely laborious, and expensive processes [6]. To cut down on the cost of drug discovery, in silico drug design and development have facilitated the discovery of alternative novel drug targets and the identification of novel drug molecules [6, 7]. Previously, Ng’ong’a et al. [8] highlighted putative molecular drug targets in *P. falciparum* for 3‐chloro‐4‐(4‐chlorophenoxy)aniline (ANI) among other potential lead compounds using cheminformatics (PubChem database) and bioinformatics (molecular docking) tools. The molecular screening revealed that ANI inhibits *P. falciparum* enoyl acyl carrier protein (ACP) reductase, the enzyme involved in the conversion of a NADH‐dependent reduction of trans‐2‐enoyl‐ACP to acyl‐ACP in the final step of the fatty acid type II biosynthesis pathway, which is present in the parasite but absent in the human host cells [8]. Since the rapidly growing malaria parasites within the human red blood cell exhibit increased lipid biosynthesis during malaria infection [9, 10], the step catalyzed by *P. falciparum* enoyl ACP reductase offers an exclusive target for new antimalarial drugs. Therefore, to verify the predictions by Ng’ong’a et al. [8], Sifuna et al. (2019) demonstrated the synergistic antimalarial activities of ANI in combination with artesunate and chloroquine in malaria parasite cultures and in mice [11]. On the other hand, Waithera et al. highlighted the antimalarial efficacy of the artesunate‐3‐chloro‐4‐(4‐chlorophenoxy)aniline hybrid drug, which was synthesized by covalently linking the pharmacophores of artesunate (ATS) and ANI [2]. However, in both studies, the safety of ANI in mammalian cells and its pharmacokinetic (PK) profile, which may provide valuable insights to facilitate further studies and drug development, were not assessed.

Profiling the PK properties as characterized by absorption, distribution, metabolism, excretion (ADME), and toxicity (Tox) of novel compounds is a critical step in the drug development process since more than 50% of novel prospective candidate drugs are discarded along the process and thus fail to reach the market due to unfavorable PK [12, 13]. Recently, computational approaches that use different ADMET prediction systems have been adopted as alternatives for early screening of PK [14]. ADME parameters are critical since they determine the route of drug administration, dosage, and frequency necessary to obtain the optimum therapeutic outcome of a drug [14], whereas the Tox profile facilitates the elimination of potential compounds with adverse side effects early in the drug development process [15, 16]. This study predicted the PK profile of a new compound, ANI, in silico using the pkCSM online platform. In vitro antiplasmodial activity was screened against *P. falciparum* CQ‐sensitive, 3D7, and CQ‐resistant W2 strains, while in vivo antimalarial efficacy was evaluated against *P. berghei* ANKA and piperaquine‐resistant (PQ^R^) *P. berghei* parasites in mouse models employing early and established infection tests alongside the standard drug ATS.

## 2. Materials and Methods

### 2.1. Compounds

ANI and ATS were purchased from Sigma Aldrich.

### 2.2. Prediction of Physicochemical Properties

The physicochemical properties of ANI: partition coefficient (LogP), water solubility, the number of hydrogen bond acceptors (HBA), hydrogen bond donors (HBD), and rotatable bonds (RB) were predicted using the pkCSM online tool (https://structure.bioc.cam.ac.uk/pkcsm) [17].

### 2.3. Prediction of PK Profile

The canonical simplified molecular input line entry system (SMILES) of ANI and ATS were retrieved from the PubChem database (https://pubchem.ncbi.nlm.nih.gov/). The SMILES were uploaded to the pkCSM online tool (https://structure.bioc.cam.ac.uk/pkcsm) and submitted for the calculation of PK: ADME and Tox profiles [17].

### 2.4. In Vitro Cytotoxicity and Antiplasmodial Activity Assays

Vero cells were used to determine cytotoxicity in vitro. These cells are regarded as a standard mammalian model to evaluate cytotoxicity during early antimalarial screening and to estimate the selectivity index (SI) between parasite inhibition and host‐cell toxicity. In vitro cytotoxicity against Vero E6 cells and antiplasmodial activity against *P. falciparum* CQ‐sensitive, 3D7, and CQ‐resistant W2 strains were screened using the 3‐(4,5‐dimethylthiazol‐2‐yl)‐2,5‐diphenyl tetrasodium bromide (MTT) colorimetric and radiolabeled ^3^[H]‐hypoxanthine incorporation assays, respectively, as described previously by Waithera et al. with slight modifications [2]. The drug solutions were prepared separately by dissolving ATS in distilled water. In contrast, ANI was dissolved in 0.5 μL DMSO, and the required initial volume was constituted by adding distilled water. The initial drug concentrations for cytotoxicity and antiplasmodial drug screening assays were 2.5 × 10^3^ μM and 4.0 × 10^0^ μM, respectively. The SI was determined by a ratio of cytotoxicity against antiplasmodial activity as described by Waithera et al. [2].

### 2.5. Early Infection Tests

The percentage parasite reduction (% PR) in early infection was evaluated using the 4‐day tests (4‐DTs) as previously described by Waithera et al. [2]. Two groups, each composed of 15 Swiss albino mice (23 g), were randomly distributed and inoculated with 1 × 10^7^ *Plasmodium berghei* ANKA or PQ^R^ *P. berghei* parasites intraperitoneally. Each parasite group was divided into two tests and a control group of five mice. After 4 h, 24 h, 48 h, and 72 h later, ANI and ATS solutions were orally administered to the test mice (two groups of five mice each) at the dosages of 3.0 and 1.5 mg/kg body weight (bwt), while the control group received distilled water. On Day 4 (D4), thin blood smears were prepared by obtaining blood from the tail snips of each mouse [2]. The smears were stained with the Giemsa staining protocol and microscopically examined under oil immersion. The % PR was determined using the method described previously by Waithera et al. [3].

### 2.6. Established Infection Tests

Fifteen mice were inoculated with 1 × 10^7^ *P. berghei* ANKA parasites. After determining the parasitemia on D4, infected mice were randomly distributed into two test groups and a control group, each of five mice. The ED_50_s (3.07 and 2.81) of ANI and ATS solutions against the *P. berghei* ANKA parasites, obtained from the 4‐DTs, were administered orally to the test mice for three consecutive days, starting from D4 to D6. The control group received distilled water. Afterward, thin blood smears were prepared on D7 to D13 and stained as previously described. The percentage reduction in parasitemia was determined from the difference in parasitemia on D4 and D7 [2].

### 2.7. Ethics Statement

The animal studies were approved by the KEMRI Animal Care and Use Committee (KEMRI/ACU 01.10.2018). All experiments were conducted following the institutional laws and regulations for animal use and care.

### 2.8. Statistical Analysis

The results for in vitro cytotoxicity and antiplasmodial assays were analyzed and expressed as mean standard deviation (SD), while the in vivo data were expressed in percentages. The significance of differences of treated groups in relation to the control untreated group was determined using ANOVA. A value of *p* < 0.05 was considered statistically significant.

## 3. Results

### 3.1. Physicochemical Properties

The predicted molecular weights (MW) of ANI and ATS were 254.11 g/mol and 384.43 g/mol, respectively. ANI is lipophilic, LogP 4.36, whereas ATS is hydrophobic, LogP 2.60. The predicted number of HBA, HBD, and RB was 2, 2, and 2 for ANI and 8, 1, and 4 for ATS, respectively, as shown in Table 1.

**TABLE 1:** Physicochemical properties of 3‐chloro‐4‐(4‐chlorophynoxy)aniline (ANI).

Parameters	ANI	ATS
Chemical name	3‐Chloro‐4‐(4‐chlorophynoxy) aniline	Artesunate
Molecular formula	C_12_H_9_Cl_2_NO_3_	C_19_H_28_O_8_
Molecular weight (g/mol)	254.11	384.42
Molecular structure	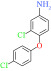	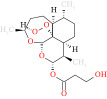
Log *p*	4.36	2.60
Water solubility	−4.36	−3.10
Number of HBA	2	8
Number of HBD	2	1
Number of RB	2	4

Abbreviations: HBA, hydrogen bond acceptors; HBD, hydrogen bond donors; Log *p*, partition coefficient; RB, rotatable bonds.

### 3.2. In Silico PK Profiles

Table 2 shows the predicted PK profile: ADME, and Tox of ANI and ATS.

**TABLE 2:** *In silico* pharmacokinetic profile of 3‐chloro‐4‐(4‐chlorophynoxy)aniline (ANI).

Parameter	ANI	ATS
*Absorption*		
HIA (% absorbed)	91.0	72.0
Caco‐2 (cm/sec)	1.1	0.86
P‐gp substrate	Substrate	Substrate
P‐gp I inhibition	Noninhibitor	Noninhibitor
Skin permeability	−2.54	−2.73

*Distribution*		
BBB permeability	0.16	−0.95
CNS permeability	−1.26	−3.03
PP (fraction unbound)	0.13	0.36

*Metabolism*		
CYP 2D6 substrate	Substrate	Nonsubstrate
CYP 3A4 substrate	Substrate	Substrate
CYP inhibition	Inhibitor	Noninhibitor
CYP 2C19 inhibition	Inhibitor	Noninhibitor
CYP 2C9 inhibition	Inhibitor	Noninhibitor
CYP 2D6 inhibition	Noninhibitor	Noninhibitor
CYP 3A4 inhibition	Noninhibitor	Noninhibitor

*Excretion*		
Total clearance	0.52	0.97
*Toxicity*		
LD_50_ (g/kg)	696.0	3.11
hERG inhibition	Non‐inhibitor	Non‐inhibitor

Abbreviations: BBB, blood–brain barrier; Caco‐2, human epithelial adenocarcinoma colorectal cell line; CYP, cytochrome P450 enzymes; hERG, human ether‐a‐go‐go‐related gene; HIA, human intestinal absorption; LD_50_, inhibitory concentration fifty in acute oral toxicity; P‐gp, P‐glycoprotein; PPB, plasma protein binding.

### 3.3. Absorption

The percentage of human intestinal absorption (% HIA), permeability to human epithelial colorectal adenocarcinoma (Caco‐2) cells, P‐glycoprotein substrate, P‐glycoprotein I (P‐gp I) inhibition, and skin permeability were used to predict the absorption profiles. ANI manifested considerably high HIA and permeability to Caco‐2 cells, 91.0% and 1.10 cm/sec, respectively, against 72.0% and 0.86 cm/sec for ATS.

ANI was predicted to be a substrate for P‐gp and a noninhibitor of P‐gp I. In addition, ANI has a negative value for skin permeability (−2.54 cm/h), similar to ATS (−2.73 cm/h), as shown in Table 2.

### 3.4. Distribution

The blood–brain barrier (BBB) penetration, central nervous system (CNS) permeability, and the unbound fraction of the drug to the plasma protein (PP) were used to predict the distribution of ANI. The predicted BBB and PP values for ANI, 0.16 C_brain_/C_blood_, and −1.26 were comparable to −0.95 C_brain_/C_blood_ and −3.03 for ATS, as shown in Table 2. However, experimental studies such as microsomal stability, PP binding, and permeability assays are required for validation.

### 3.5. Metabolism

ANI was screened for its potential of being either an inhibitor or substrate for various human hepatic cytochrome P450 (CYP) drug‐metabolizing enzymes: CYP 1A2, CYP 2C19, CYP 2C9, CYP 2D6, and CYP 3A4. The predictions revealed that ANI is a substrate and noninhibitor of CYP 2D6 and CYP 3A4 and an inhibitor of CYP 1A2, CYP 2C19, and CYP 2C9, as shown in Table 2. However, the metabolic profile obtained in this study cannot be considered conclusive without further experimental validation.

### 3.6. Excretion

The total clearance (TL) values for ANI and ATS were 0.52 log mL/min/kg and 0.97 log mL/min/kg, respectively, as shown in Table 2.

### 3.7. Tox

The predicted fifty percent inhibitory concentration in acute oral Tox (LD_50_) in rats for ANI was 696.0 g/kg against 3.11 g/kg for ATS. ANI exhibited noninhibition of the human ether‐a‐go‐go‐related gene (hERG) potassium ion (K^+^) channel, similar to ATS, as shown in Table 2.

### 3.8. In Vitro Cytotoxicity and Antiplasmodial Activity

The fifty percent cytotoxicity concentration (CC_50_) against Vero E6 cells was 7.90 × 10^2^ ± 86.40 μM and 1.88 × 10^4^ ± 19.22 μM for ANI and ATS, respectively. In contrast, the fifty percent inhibitory concentrations (IC_50_s) for *P. falciparum* against CQ‐sensitive 3D7 and CQ‐resistant W2 strains were (1.72 ± 0.09 and 1.84 ± 0.21) μM for ANI and (0.76 ± 0.35 and 0.73 ± 0.05) μM for ATS. The SI for ANI was 459 and 429 and 24,736 and 25,753 for ATS against CQ‐sensitive 3D7 and CQ‐resistant W2 strains, respectively.

The cytotoxicity assays showed that ANI and ATS exhibited markedly different safety profiles toward Vero E6 cells. The CC_50_ value for ANI was 7.90 × 10^2^ ± 86.40 μM, indicating moderate cytotoxicity, whereas ATS displayed substantially lower cytotoxicity, with a CC_50_ of 1.88 × 10^4^ ± 19.22 μM (Table 3).

**TABLE 3:** In vitro cytotoxicity against Vero E6 cells, antiplasmodial activity against *P. falciparum* CQ‐sensitive, 3D7 and CQ‐resistant W2 strains (mean ± SD), and selectivity indices of 3‐chloro‐4‐(4‐chlorophynoxy)aniline (ANI).

Parameter	CC_50_ mean ± SD (Vero E6 cells)	IC_50_ mean ± SD (3D7)	IC_50_ mean ± SD (W2)	SI (3D7)	SI (W2)
ANI	7.90 × 10^2^ ± 86.40 μM	1.72 ± 0.09 μM	1.84 ± 0.21 μM	459	429
ATS	1.88 × 10^4^ ± 19.22 μM	0.76 ± 0.35 μM	0.73 ± 0.05 μM	24,736	25,753

ANI demonstrated moderate antiplasmodial activity against *P. falciparum* with IC_50_ values of 1.72 ± 0.09 μM and 1.84 ± 0.21 μM against the CQ‐sensitive 3D7 and CQ‐resistant W2 strains, respectively (Table 3). ATS showed even greater potency, with IC_50_ values of 0.76 ± 0.35 μM (3D7) and 0.73 ± 0.05 μM (W2) (Figure 1(b)). The comparable IC_50_ values between the two isolates also indicate that both ANI and ATS maintain antiplasmodial activity against CQ‐resistant parasites, suggesting their potential to overcome CQ resistance.

FIGURE 1Antimalarial efficacy of ANI against *P. berghei* ANKA and *P. berghei* piperaquine‐resistant (PQ^R^) parasites in mouse models in an early infection test: (a) percentage parasitemia reduction and (b) fifty percent *in vivo* inhibitory concentrations (ED_50_s) against *P. berghei* ANKA and PQ^R^ parasites.

**Figure:**
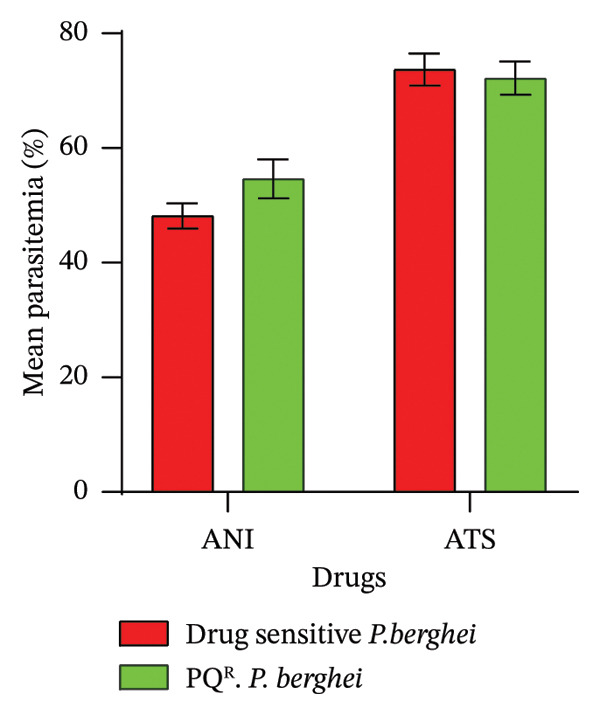
(a)

**Figure:**
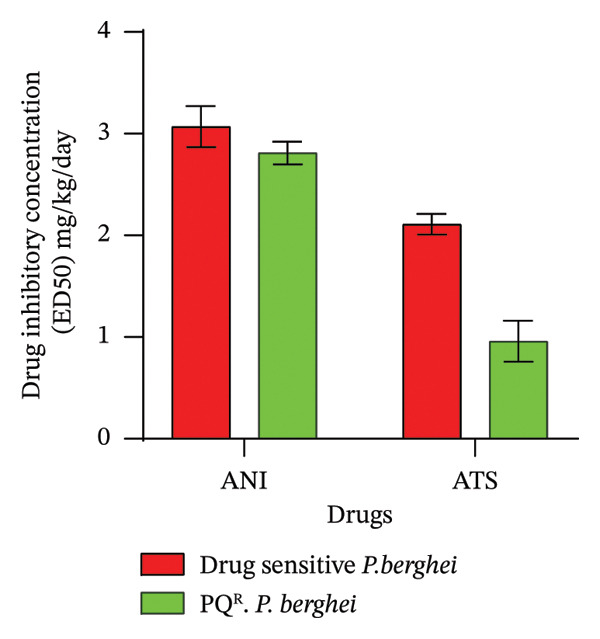
(b)

Calculation of the SI further highlights the broad safety margin of ANI, exhibiting SI values of 459 (3D7) and 429 (W2) even though the reference drug is ATS. Even though ATS displayed substantially higher SI values of 24,736 (3D7) and 25,753 (W2) compared to ANI, the SI of greater than 400 for ANI indicates a broad safety margin, making it a promising new compound for further drug development.

### 3.9. Early Infection Tests

ANI exhibited moderate but consistent antimalarial activity against both *P. berghei* ANKA and PQ^R^ *P. berghei* parasites. Figure 1(a) shows the % PR (48.19% and 54.66%) mg/kg/bwt for ANI and (73.69% and 72.17%) mg/kg/bwt for ATS against *P. berghei* ANKA and PQ^R^ *P. berghei* parasites, respectively. Figure 1(b) shows the ED_50_s (3.07 and 2.81 mg/kg/day) for ANI and (2.81 and 0.96) mg/kg/day for ATS against *P. berghei* ANKA and PQ^R^ *P. berghei* parasites, respectively. Although ATS remains more potent, the ability of ANI to achieve substantial parasitemia reduction and similar ED_50_ values against both sensitive and resistant parasites highlight its potential as a promising antimalarial compound. The preserved activity of ANI against PQ^R^ parasites suggests a possible distinct or resistance‐breaking mechanism of action.

### 3.10. Established Infection Tests

**FIGURE 2:**
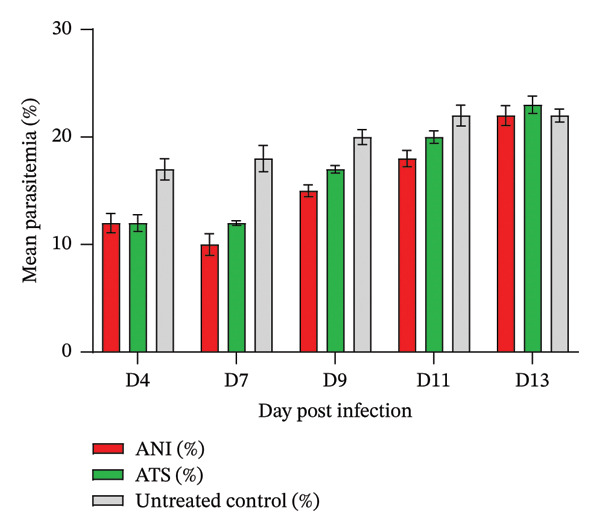
Antimalarial efficacy of ANI against *P. berghei* ANKA parasites in mouse model. Infected mice were treated from D4 to D6. Smears were made on D7 to D13. Parasitemia level reduced in the test groups compared to the untreated control on D7, *p* > 0.05, and gradually increased from D9 to D13.

The mice treated with either ANI or ATS showed a reduction in % parasitemia relative to the untreated control mice, which was not statistically significant (*p* > 0.05) at the dosage tested (Figure 2). On Day 4 (D4), parasitemia levels were comparable across groups, with ANI and ATS both at 12.0%, and the untreated control slightly higher at 13.0%. By Day 9 (D9), clear differences emerged: parasitemia rose to 15.0% in the ANI‐treated group and 17.0% in the ATS‐treated group, while the control group exhibited a higher parasitemia level of 19.0%. Overall, the results suggest ANI and ATS exert suppressive effects on malaria parasites’ proliferation, particularly during midstage infection (D9). The ability of ANI to maintain parasitemia at levels comparable to, or slightly lower than, ATS at later stages indicates promising in vivo antimalarial activity. Notably, all the mice treated with ANI survived for 30 days with no observable physical change. This supports ANI as a potential new compound to be considered for further antimalarial development. Continued evaluation, including dose optimization and PK studies in vivo, is warranted to determine whether ANI can provide sustained parasite suppression and improved survival outcomes relative to standard therapies.

## 4. Discussion

The demand for alternative malaria medicine is a global priority, particularly in malaria‐endemic countries of Southeast Asia and Sub‐Saharan Africa, continents experiencing increased cases of declining efficacy of the ACT against the human malaria parasites. The current pressing situation led to the discovery of a new potential antimalarial compound, ANI, using cheminformatics (PubChem) and bioinformatics (molecular docking) tools. The present study predicted the physicochemical and PK (ADMETox), profile in silico; screened its cytotoxicity in Vero E6 cells and antiplasmodial activity against *P. falciparum* CQ‐sensitive 3D7 and CQ‐resistant W2 strains; and evaluated antimalarial in vivo efficacy against *P. berghei* ANKA and PQ^R^ *P. berghei* parasites in mouse models employing early and established infection tests alongside the standard drug ATS.

*In silico* screening for the PK (ADMETox) profile of novel molecules and prospective drug candidates provides insights into whether to proceed with in vitro or in vivo PK studies or eliminate potent compounds that are likely to cause adverse clinical outcomes, thus saving time and production costs [15]. PK of ANI was screened based on the ADMETox profile, parameters that influence drug disposition, and therapeutic outcomes [18].

Absorption is a key determinant of the bioavailability of orally administered antimalarial drugs. *In silico* absorption properties were assessed by predicting HIA (%), permeability to Caco‐2 cells, P‐glycoprotein substrate, and P‐glycoprotein I (P‐gp I) inhibition when administered orally, whereas skin permeability was used to predict the possibility of ANI and its formulations for transdermal delivery. ANI manifested a considerably higher HIA, 91% against 72% for ATS. The Caco‐2 cells are commonly used to estimate the ability of a compound to diffuse through the human gastrointestinal tract (GIT) compared to the human enterocytes, a vital aspect for determining drug metabolism [19]. The permeability of ANI (1.0 cm/sec) to Caco‐2 cells was similar to that of ATS (0.86 cm/sec). P‐gp, also known as multidrug resistance protein‐1 (MDR1), is a member of the ABC transmembrane protein family [16, 20], which is involved in the active transport of drugs across the plasma membrane [16, 21]. The degree of affinity of drugs to P‐gp influences oral bioavailability [20] and PK profiles [16]. ANI was predicted to be a noninhibitor of P‐gp I, which suggests that maximum drug efficacy outcomes will be attained since most of the active components of the drug will reach the intended sites of action [16]. Transdermal delivery of drugs provides a promising non‐invasive and affordable alternative to conventional oral drug administration [22, 23]. For instance, a study by Zech et al. demonstrated an enhanced efficacy of artemisone, a derivative of artemisinin, when its microemulsion formulation was given through transdermal delivery [24]. The negative skin permeability values exhibited by ANI, which were similar to those of ATS, suggest that ANI is impermeable through the skin [2] and might be unsuitable for transdermal administration [19].

Drug distribution was predicted by screening for the permeability of ANI across the BBB and the CNS and its affinity to the PP. According to Stéen et al., for compounds to be active in the CNS, they have to satisfy the following physicochemical endpoints: Log *p* > 0.40, HBA ≤ 4, and RB ≤ 7 [25]. The predicted CNS value for ANI met this criterion, suggesting its potential for broad therapeutic use once its safety is substantiated.

The human hepatic cytochrome P450 (CYP) enzyme families: CYP families CYP1, CYP2, and CYP3 play a vital role in the biotransformation of most clinically active drugs [26]. The process involves degradation of drugs by enzymes via the phase I reactions to facilitate their final elimination [18] from the systemic circulation in the form of urine or bile [26]. Drug metabolism also determines the dosage and frequency of drug administration and selection for drug resistance. Genetic polymorphism in the CYP 450 genes significantly influences [26] interpatient treatment variability, especially in individuals from different ethnic backgrounds. CYP450 drug substrates or inhibitors also influence drug–drug interaction for combination therapies or coadministered drugs, especially in patients with other co‐morbidities. ANI is a substrate of CYP 3A4 and CYP 2D6, two key CYP 450 enzymes involved in the metabolism of most drugs, revealing its potential for effective metabolism and efficient elimination from the system. However, predicted inhibition of CYP1A2, CYP2C19, and CYP2C9 may indicate potential drug–drug interaction risks. Therefore, there is need for further experimental validation for the predicted metabolic profile to be considered definitively favorable.

Tox studies facilitate the elimination of potential compounds that exhibit adverse side effects early in the drug development process. Tox was determined by predicting the fifty percent inhibitory concentration in acute oral toxicity (LD_50_), and the hERG potassium ion (K^+^) channel inhibition risks. The predicted LD_50_ in rats for ANI, 696.0 mg/kg, suggests that it possesses a broad therapeutic index, > 50 times the ED_50_ (3.07 mg/kg/day), consistent with the broad in vitro SI. Inhibition of the hERG K^+^ channel by novel drug candidates indicates their potential to cause drug‐induced QT interval prolongation, which is a high‐risk factor associated with chronic arrhythmias [2, 15] known as Torsade’s de pointes [27, 28] and sudden cardiac‐related deaths [29]. ANI exhibited a noninhibition of the hERG K^+^ channel, a profile comparable to ATS, suggesting that it would exhibit minimum adverse cardiovascular risks.

Although in silico characterization of the PK profile reduces the time and financial liabilities of the drug development process, further validation using animal models is necessary. The present study gives vital insights into the PK of ANI. Therefore, it is important to use animal models to validate the oral bioavailability of the predicted high HIA, 91.0% of ANI, and assess the hERG K^+^ channel inhibition risks.

The CC_50_ against the Vero E6 cells was 7.90 × 10^2^ ± 86.40 μM and 1.88 × 10^4^ ± 19.22 μM for ANI and ATS, respectively. Notably, safety evaluation in hepatocyte and cardiomyocyte models, as well as erythrocyte hemolysis tests, is necessary to further assess the safety of ANI. On the other hand, the IC_50_s against *P. falciparum* CQ‐sensitive 3D7 and CQ‐resistant W2 strains were 1.72 ± 0.09 and 1.84 ± 0.21 μM and 0.76 ± 0.35 and 0.73 ± 0.05 μM for ANI and ATS, respectively. The SI was 459 and 429 for ANI and 24,736 and 25,753 for ATS against *P. falciparum* CQ‐sensitive 3D7 and CQ‐resistant W2 strains, respectively, which translates to minimum off‐target effects on the human host. Recent studies have shown that varying the in vitro antiplasmodial screening assay time from the standard 2‐day (2‐D) to 4‐D or 6‐D resulted in improved efficacy of the test drugs alstonine and himbeline and eravacycline, respectively, against the *P. falciparum* CQ‐sensitive 3D7 strain [4, 5]. It would be interesting to screen the antiplasmodial activity of ANI using the extended‐time assay protocols to determine its mode of action and assess any improvement in its antiplasmodial activity.

In the early infection test, the percentage reduction in parasitemia and ED_50_ for ANI were 48.19% and 54.66% mg/kg/bwt and 3.07 and 2.81 mg/kg/day against *P. berghei* ANKA and PQ^R^ *P. berghei* parasites, respectively. On the other hand, the reference drug ATS exhibited a chemosuppression of 73.69% and 72.17% mg/kg/bwt and ED_50_s of 2.1 and 0.96 mg/kg/day against *P. berghei* ANKA and PQ^R^ *P. berghei* parasites, respectively. Although ANI shows lower IC_50_ and ED_50_ compared to the reference drug ATS, combining ANI with a long‐acting antimalarial drug, such as chloroquine, in line with the WHO recommendation for malaria treatment, provided an overall increase in antimalarial efficacy [11]. Therefore, other drug combinations can be explored to determine the prospective scope of ANI for malaria treatment. In addition, in vivo antimalarial efficacy used other resistant parasites (amodiaquine, lumefantrine, and pyronaridine).

In the established infection test, a remarkable reduction in parasitemia was observed in mice treated with either ANI or ATS relative to the untreated control group when the percentage parasitemia was compared between D4 and D7, even though the parasitemia gradually increased from D9 to D13 in both the treated and untreated groups. Most importantly, all the mice treated with ANI survived for 30 days without physical changes indicating sustained parasite clearance over time. Overall, since the molecular target of ANI remains invalidated, further studies—including enzyme inhibition assays, metabolomics, and genetic validation—are required to confirm its mechanism of action. ANI should therefore be considered an early‐stage phenotypic hit warranting additional optimization and resistance profiling.

## 5. Conclusions

*In silico* predictions show that ANI is a substrate for CYP 3A4 and CYP 2D6 and is unlikely to inhibit the hERG potassium ion channel. ANI exhibited a low cytotoxicity profile, with a CC_50_ value of 7.90 × 10^2^ ± 86.40 μM, and potent antiplasmodial activity, with IC_50_ values of 1.72 ± 0.09 μM and 1.84 ± 0.21 μM against the *P. falciparum* CQ‐sensitive 3D7 and CQ‐resistant W2 strains, respectively. The SI of greater than 400 indicates that ANI has a broad safety margin. In vivo, ANI demonstrated antimalarial efficacy with ED_50_ values of 3.07 mg/kg/day against *P. berghei* ANKA and 2.81 mg/kg/day against PQ^R^ *P. berghei* parasites. Notably, mice treated with ANI in the established infection test survived for 30 days without physical changes. The rapid parasiticidal action and sustained in vivo suppression highlight ANI as a potential antimalarial compound and support its further drug evaluation and development.

## Author Contributions

Milka W. Waithera: funding acquisition, project management, in vitro and in vivo efficacy studies, in silico data acquisition and interpretation, drafting of the original manuscript, editing, and revision. Shadrack K. Kimani and Johnson K. Kinyua: manuscript review.

## Funding

Milka W. Waithera was financially supported by the National Commission for Science and Technology and Innovation (NACOSTI/RCD/ST&I 6th CALL MSc 055), Kenya.

## Conflicts of Interest

The authors declare no conflicts of interest.

## Data Availability

Data will be made available from authors on request.
